# Raised intracranial pressure alters cortical vascular function and cephalic allodynia

**DOI:** 10.1093/brain/awae415

**Published:** 2025-03-08

**Authors:** Olivia Grech, Eloisa Rubio-Beltran, Emily C Stanyer, Alejandro Labastida-Ramirez, Gareth G Lavery, Lisa J Hill, Philip R Holland, Alexandra J Sinclair

**Affiliations:** Metabolism and Systems Science, College of Medicine and Health, University of Birmingham, Birmingham B15 2TT, UK; Biomedical Sciences, College of Medicine and Health, University of Birmingham, Birmingham B15 2TT, UK; Headache Group, Wolfson Sensory, Pain and Regeneration Centre, Institute of Psychiatry, Psychology and Neuroscience, King’s College London, London SE1 3RB, UK; Headache Group, Wolfson Sensory, Pain and Regeneration Centre, Institute of Psychiatry, Psychology and Neuroscience, King’s College London, London SE1 3RB, UK; Sleep and Circadian Neuroscience Institute, Nuffield Department of Clinical Neurosciences, University of Oxford, Oxford OX3 9DU, UK; Headache Group, Wolfson Sensory, Pain and Regeneration Centre, Institute of Psychiatry, Psychology and Neuroscience, King’s College London, London SE1 3RB, UK; Centre for Systems Health and Integrated Metabolic Research, Department of Biosciences, School of Science and Technology, Nottingham Trent University, Clifton Campus, Nottingham NG11 8NS, UK; Metabolism and Systems Science, College of Medicine and Health, University of Birmingham, Birmingham B15 2TT, UK; Biomedical Sciences, College of Medicine and Health, University of Birmingham, Birmingham B15 2TT, UK; Headache Group, Wolfson Sensory, Pain and Regeneration Centre, Institute of Psychiatry, Psychology and Neuroscience, King’s College London, London SE1 3RB, UK; Metabolism and Systems Science, College of Medicine and Health, University of Birmingham, Birmingham B15 2TT, UK

**Keywords:** calcitonin gene-related peptide receptor antagonist, olcegepant, glucagon-like peptide receptor agonist, exenatide

## Abstract

Raised intracranial pressure (ICP) is associated with altered cerebral haemodynamics and cephalic pain. The relationship between the algetic response and cortical neurovascular changes in raised ICP is unclear. This study aimed to evaluate this relationship and determine whether lowering ICP (using a glucagon-like peptide-1 receptor agonist) could ameliorate the algetic response. We also sought to explore the role of calcitonin gene-related peptide in cephalic pain driven by raised ICP by inhibiting calcitonin gene-related peptide signalling and quantifying changes in the algetic response.

In a rat model of raised ICP, created by intracisternal kaolin injection, mechanical thresholds were measured alongside steady-state potential and cerebral blood flow responses to spreading depolarization. Nuclear magnetic resonance spectroscopy evaluated energetic substrates in animals with raised ICP *ex vivo*. The glucagon-like peptide-1 receptor (GLP-1R) agonist exenatide and the calcitonin gene-related peptide receptor (CGRP-R) antagonist olcegepant were injected daily, and measurements were repeated.

Kaolin increased ICP [median (range) 15.96 (8.97) mmHg, *n* = 8] versus controls [6.02 (1.79) mmHg, *n* = 6, *P* = 0.0007]. Animals with raised ICP exhibited reduced mechanical thresholds [mean (standard deviation) hind paw baseline: 5.78 (2.81) g, Day 7: 3.34 (2.22) g, *P* < 0.001; periorbital baseline: 6.13 (2.07) g, Day 7: 2.35 (1.91) g, *n* = 12, *P* < 0.001]. Depolarization and repolarization durations were increased [depolarization, raised ICP: 108.81 (222.12) s, *n* = 11, controls: 37.54 (108.38) s, *n* = 9, *P* = 0.038; repolarization, raised ICP: 1824.26 (3499.54) s, *n* = 12, controls: 86.96 (140.05) s, *n* = 9, *P* < 0.0001]. Cerebral blood flow change was also reduced [85.55 (30.84)%, *n* = 9] compared with controls [217.64 (37.70)%, *n* = 8, *P* < 0.0001]. Substrates for cellular energetics (ADP, ATP and NAD^+^) were depleted in rodent brains with raised ICP (*P* = 0.009, *P =* 0.018 and *P* = 0.011, respectively).

Exenatide significantly lowered ICP [exenatide: 9.74 (6.09) mmHg, *n* = 19, vehicle: 18.27 (6.67) mmHg, *n* = 16, *P* = 0.004] and rescued changes in mechanical withdrawal. Exenatide recovered characteristic spreading depolarization responses [depolarization duration, exenatide: 56.46 (25.10) s, *n* = 7, vehicle: 115.98 (58.80) s, *n* = 6, *P* = 0.033; repolarization duration, exenatide: 177.55 (562.88) s, *n* = 7, vehicle: 800.85 (1988.67) s, *n* = 6, *P* = 0.002]. In the setting of raised ICP, olcegepant prevented changes in periorbital mechanical thresholds.

We conclude that raised ICP disrupted the cortical neurovascular responses, reduced algetic thresholds and depleted crucial energetic substrates. Exenatide reduced ICP, improving algetic thresholds and cortical neurovascular changes. Importantly, olcegepant alleviated the cerebral algesia, suggesting a role for calcitonin gene-related peptide in driving pain responses in elevated ICP.

These studies support the rationale that reducing ICP improves cephalic pain in conditions of raised ICP. Furthermore, the data suggest that headache pain in diseases associated with raised ICP could be ameliorated therapeutically though blockade of the calcitonin gene-related peptide pathway.

## Teaser

Raised intracranial pressure (ICP) is associated with altered cerebral blood flow and cephalic pain. In a rat model, Grech *et al.* show that a GLP-1R agonist reduced ICP and increased pain thresholds, while blocking CGRP signalling reduced cephalic pain. These findings could lead to new treatments for headaches linked to raised ICP.

## Introduction

Raised intracranial pressure (ICP) is associated with altered cerebral haemodynamics^1,2^ and cephalic pain.^3,4^ Cortical vascular change, specifically spreading depolarization, is a key indicator of altered excitability in neurological conditions featuring raised ICP, including traumatic brain injury,^5^ subarachnoid haemorrhage^6^ and stroke.^7^ Conditions of raised ICP are known to generate cephalic pain.^3,4,8^ However, the relationships between raised ICP, the extent of cortical neurovascular changes and the degree of allodynia are not understood.

Cerebral allodynia is associated with the peptide calcitonin gene-related peptide (CGRP) in migraine and traumatic brain injury.^9,10^ Levels are elevated during migraine attacks,^11^ and infusion of CGRP can provoke a migraine-like attack in migraine patients but not in controls.^12^ Attenuation of CGRP is a therapeutic strategy currently licensed for migraine.^13-16^ Studies have shown an association between CGRP monoclonal antibodies and reduction in headache in idiopathic intracranial hypertension,^17,18^ and post-traumatic headache.^19^ However, the relationship between cerebral allodynia, CGRP and raised ICP has not previously been explored. Human studies have demonstrated markedly disrupted metabolism in the CNS of patients with raised ICP^20^ and the association between disordered metabolism and headache risk.^21^ Characterizing cerebral energetics demands in raised ICP, however, has not been explored previously.

To probe relationships between raised ICP, cerebral vascular responses and cephalic pain, we used a validated rat model of raised ICP, in which we evaluated cortical neurovascular changes, including spreading depolarization and cerebral blood flow (CBF), in addition to cephalic pain quantified by mechanical withdrawal thresholds. We then aimed to abrogate this model by reducing ICP using a glucagon-like peptide 1 receptor (GLP-1R) agonist, which has been shown in animal models and clinical trials to reduce ICP.^22,23^ Finally, we aimed to explore the role of CGRP in the cephalic pain driven by raised ICP by inhibiting CGRP and quantifying changes in the algetic response.

## Materials and methods

### Animal husbandry

Male Sprague–Dawley rats (*n =* 140; Enivgo) aged 9–21 weeks were used. Only male animals were used to avoid the well-known interaction of cycling sex hormones with ICP and spreading depolarization responses.^24-26^ Rats were housed in litter-matched groups in a climate-controlled room and kept on a 12 h–12 h light–dark cycle with free access to food and water. All animal handling and procedures were performed according to the guidelines specified by the UK Home Office and Animals (Scientific Procedures) Act 1986 and approved by the King’s College London Animal Welfare and Ethical Review Body (PP9890223, approved in September 2022). Animal studies are reported in compliance with ARRIVE 2.0 guidelines.

### Raised intracranial rodent model

**Figure 1:**
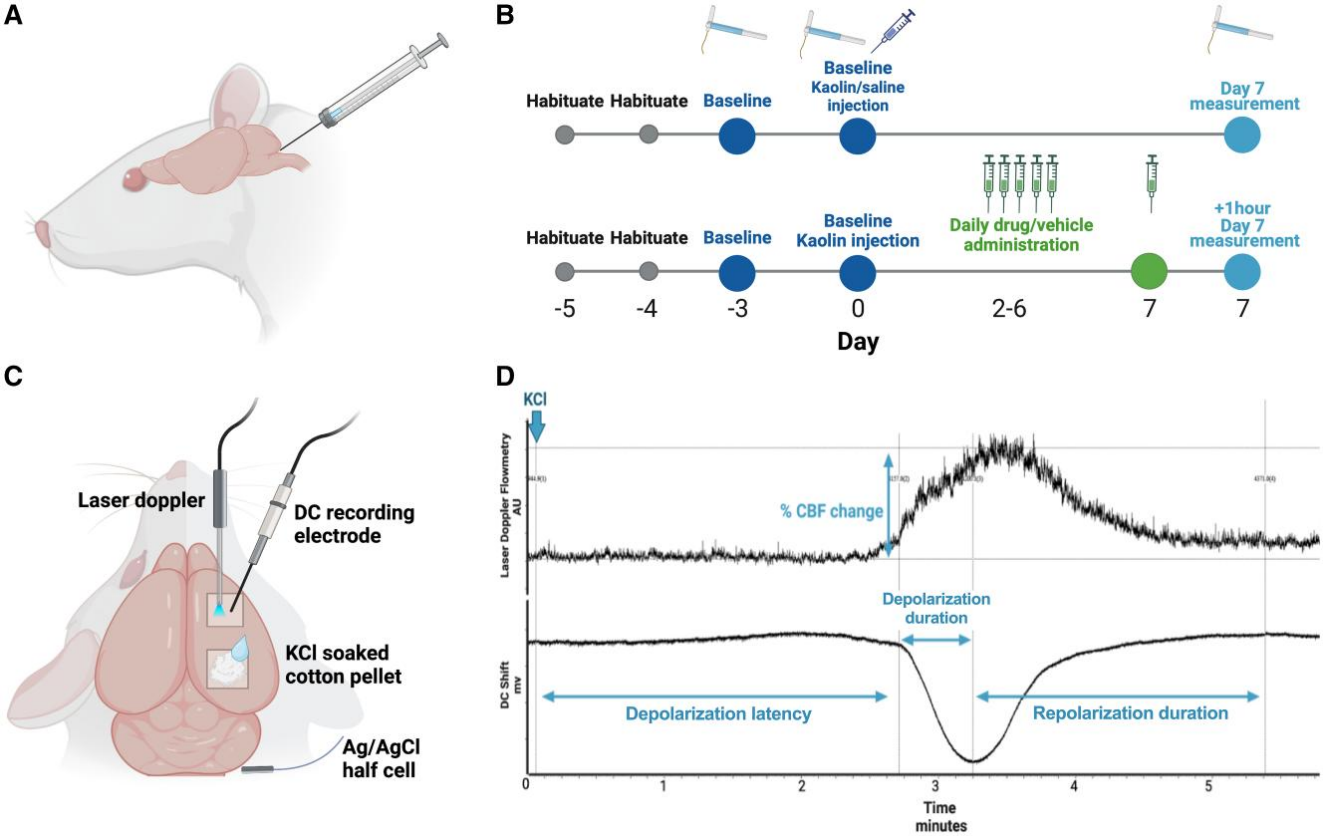
**Methodology of the raised intracranial pressure rat model and assessments.** (**A**) Kaolin or an equal volume of saline (control) was injected into the cisterna magna of rats percutaneously. (**B**) Parameters calculated from the first depolarization event following stimulation. (**C**) Surgical preparation of spreading depolarization assessments, which measured cerebral blood flow and steady-state potential (direct current, DC) changes during evoked spreading depolarization using a KCl-soaked pellet on the cortex. (**D**) Time line of mechanical withdrawal threshold assessments in animals with raised intracranial pressure versus controls, and of drug versus vehicle. AU = arbitrary units. Created in BioRender.

A validated rat model of raised ICP was used, which uses injection of kaolin suspension into the cisterna magna to induce obstructive hydrocephalus and thereby increase ICP.^23,27^ Under isoflurane (IsoFlo 5%, Zoetis) gaseous anaesthesia, the posterior of the head of the animal was shaved and the neck flexed to maximize foramen magnum exposure (Fig. 1A). A 30-gauge insulin needle was used to inject percutaneously 40 μl of 250 mg/ml kaolin suspension or an equal volume of 0.9% saline into the cisterna magna (determined by pilot experiments and in conjunction with literature of validated hydrocephalus models^28^). All animals were then injected subcutaneously with 5 mg/kg carprofen for analgesia and 1 ml/100 g of 0.9% saline to prevent dehydration and placed in a heated recovery chamber. Animals were weighed and monitored daily, and analgesia was administered as needed. For those used in mechanical withdrawal threshold testing, administration of analgesia was matched between groups and never within 72 h of the time of assessment. For those used in analysis of spreading depolarization, analgesia was never given within 48 h of the time of assessment.

### Animal surgery for spreading depolarization and intracranial pressure measures

To measure spreading depolarization responses and ICP, rats were anaesthetized and surgically prepared as follows. Rats were anaesthetized with isoflurane gas (5% in 0.1% oxygen–0.3% air mix) and maintained via a mask at 2% isoflurane. The femoral vein was cannulated to maintain anaesthesia with intravenous propofol (PropoFlo Plus, 30–50 mg/kg/h, Zoetis). Rats were also tracheotomized to allow ventilation with oxygen-enriched air and maintenance of the tidal CO_2_ (3.5–4.5%). Animals were maintained on propofol anaesthesia for the duration of ICP and spreading depolarization recordings. Body temperature was monitored and maintained at 36.5–37°C and the head of the animal was fixed in a stereotactic frame (Kopf Instruments).

### Intracranial pressure measurements

A solid-state ICP catheter (Millar) was used to measure the epidural ICP of rats. The catheter was connected to a single-channel bridge amplifier with a catheter interface cable (AD Instruments). Data were acquired using a Powerlab 4SP (AD Instruments) and recorded and analysed using Labchart software (Labchart 7 v.7.3.8, 24 August 2016, AD Instruments). The catheter was calibrated using a pressure gauge kit (AD Instruments) prior to use. A small hole was drilled into the parietal bone of the skull and the ICP catheter was slowly introduced into the epidural space and secured with Bonewax (Ethicon). Compression of the jugular vein and the corresponding increase in pressure was used to confirm correct insertion. The recording was allowed to settle until a consistent signal was obtained and the mean of a 5 min recording was calculated using the Labchart software.

### Electrophysiological recording of spreading depolarization responses

To measure spreading depolarization responses, two hemi-cranial windows were drilled into the parietal bone of the skull using a saline-cooled drill (Fig. 1C). A glass microelectrode (tip diameter 5 μm) filled with 3 M NaCl, was placed 800 μm below the cortical surface to allow for cortical steady-state potential recordings [direct current (DC) shift]. The microelectrode was coupled to an Ag/AgCl pellet and a reference Ag/AgCl electrode placed on the neck. The signal was fed through a DC preamplifier (NL102, gain ×1000, Neurolog, Digitimer), filtered (N125, Neurolog, Digitimer), then passed through a second-stage amplifier (NL106, Neurolog, Digitimer), through an analog-to-digital converter (Power 1401plus, CED) and displayed on a personal computer, where it was processed and stored (Spike5 v.8.04, CED). CBF was additionally monitored via laser Doppler (Moor Instruments) using a probe that was placed on the cortical surface parallel with the microelectrode.

The cortex was left to rest for ∼30 min after any potential activity induced by the electrode placements, during which a stable baseline recording was attained. As previously described,^29^ a cotton pellet soaked in 1 M KCl was subsequently placed in the posterior cranial window and the shift in DC currents was recorded. Five microlitres of 1 M KCl was added to the pellet every 15 min for 1 h. CBF and steady-state potential responses were measured using the following parameters: depolarization latency [time (in seconds) between KCl application and depolarization induction]; depolarization duration [time (in seconds) between the start of the depolarization and the most negative apex]; repolarization duration [time (in seconds) between most negative apex and return to DC baseline]; and percentage CBF change [(peak CBF response during spreading depolarization minus the baseline CBF)/baseline CBF × 100; Fig. 1B]. The number of CBF peaks coupled to depolarization events was also counted and displayed as whole numbers.

### Von Frey assessment of mechanical thresholds

To determine changes in measures of allodynia, hind paw and periorbital mechanical withdrawal thresholds were assessed. The von Frey assay was used, with calibrated von Frey filaments and the up–down method, to determine the 50% mechanical withdrawal threshold.^30^ Rats were habituated to the testing apparatus [individual ventilated acrylic enclosures on a perforated metal platform (Ugo Basile)] on two prior occasions and 1 h before the assessment (total of three habituations). The room was maintained between 30 and 50 lux and at ∼21°C, and testing was conducted at the same time (within 1 h) each day to avoid circadian variation. The same experimenter conducted measurements and was blinded to group allocation. Filaments of increasing weight were applied perpendicular to the periorbital and hind paw region of the rat, with the aim to elicit a positive response (flinching away from the filament, burrowing of the head or repeated grooming). Baseline measurements were repeated twice and averaged and measured once on Day 7 after cisterna magna injection (Fig. 1D).

### Therapeutic agents

The GLP-1R agonist exenatide (Byetta, AstraZeneca) was used to investigate the impact of lowering ICP. One day after cisterna magna injection, all animals were given analgesia (5 mg/kg carprofen). On Days 2–6, animals were injected daily with 20 μg/kg exenatide (clinical dose^22,23^) or an equal volume of saline subcutaneously. On the final day, exenatide or vehicle was administered 1 h before end-point experiments (mechanical threshold or cortical response testing).

The CGRP receptor (CGRP-R) antagonist olcegepant (BIBN4096BS, Sigma) was used to explore the impact of inhibiting CGRP-R signalling in rats with raised ICP. One day after cisterna magna injection, all animals were given analgesia (5 mg/kg carprofen). On Days 2–6, animals were injected daily with 1 mg/kg olcegepant (with 0.02% dimethyl sulphoxide) or an equal volume 0.02% dimethyl sulphoxide intraperitoneally. Dosage was determined based on the available literature demonstrating biological effects in rodents.^31,32^ On the final day, olcegepant or vehicle was administered 1 h before end-point experiments.

### Measurements of ventricular dilatation

Following assessments, animals were perfused transcardially with 1 ml/g bodyweight of 0.01 M heparinized PBS and 4% paraformaldehyde. Coronal slices 1 mm thick were made using a large rat brain matrix and imaged using a flatbed scanner (Epson Perfection V7000 Photo). Images were analysed on ImageJ Fiji, and the ventricle:brain ratio was calculated using (*a* + *b*/*c*), in which *a* and *b* denote the widths of the lateral ventricles, and *c* denotes the width of the entire brain slice across the ventricles (Supplementary Fig. 1A).

### Metabolomics

#### Metabolite extraction

Metabolites from whole-brain samples were extracted before processing. In brief, methanol (−80°C) was added to all samples to quench metabolism. Samples were then incubated on wet ice, and chloroform was added to aid the separation of non-polar metabolites. This mixture was vortexed and centrifuged at 4°C to enable separation of polar metabolites. Two millilitres of the polar fraction was transferred to a new tube and dried. The polar phase was then reconstituted in sodium phosphate buffer (100 mM sodium phosphate, 500 μM 4,4-dimethyl-4-silapentane-1-sulfonic acid and 2 mM imidazole in 100% deuterium oxide).

#### Nuclear magnetic resonance spectroscopy

^1^H-Nuclear magnetic resonance spectra were obtained from all samples using a 600 MHz Bruker Avance III spectrometer with a 1.7 mm z-PFG TCI Cryoprobe at 300 K. Solvent suppression was achieved using the NOESY presaturation pulse sequence. Spectral width was set to 12.2 ppm, and 16 384 complex data points were acquired with a 4.0 s interscan relaxation delay. All nuclear magnetic resonance spectra were processed using the MetaboLab software.^19^ Free induction decays were apodized using an exponential line broadening of 0.3 Hz and zero-filled to 131 072 real data points before Fourier transformation. The sodium trimethylsilylpropanesulfonate (DSS) internal standard signal in each spectrum was referenced to 0.0 ppm, followed by manual phase correction and batch baseline correction using a spline baseline before export into Bruker format. The area under each peak of interest was integrated and compared with the trimethylsilylpropanoic acid peak to calculate the millimolar concentration. Dried tissue weight was used for normalization.

### Statistical analysis

Sample sizes were calculated using statistical power calculations based on previous pilot data and published studies^23,33,34^ and considered 10% failure rate of kaolin injection. All statistical analysis and graphs were made using Graphpad Prism v.8 (GraphPad Software, San Diego, CA, USA). The normality of data was assessed using the Shapiro–Wilk test. Data that were normally distributed were analysed using parametric tests (*t*-tests or Pearson’s correlation coefficient) and reported as the mean and standard deviation (SD). Non-normally distributed data were analysed using non-parametric tests (Mann–Whitney U-test or Spearman rank correlation test) and reported as the [median (range)]. Confidence intervals (CI) are also reported for non-parametric data. Results were considered statistically significant when *P*-values were: *P < 0.05, **P < 0.01, ***P < 0.001 and ****P < 0.0001.

## Results

### Optimization of the kaolin model of raised intracranial pressure

Pilot studies aimed to optimize the volume of a kaolin suspension (40, 70 and 80 μl) to induce effects of moderate hydrocephalus. Only 40 μl resulted in a significantly higher ventricle:brain ratio than saline-injected controls [mean (SD) ratio, 40 µl saline: 0.01 (0.07) *n* = 6, 40 µl kaolin: 0.14 (0.07) *n* = 9, *P* = 0.008; Supplementary Fig. 1B and C]. Those injected with 40 μl of kaolin showed minimal weight loss with milder side effects, and 40 μl was therefore chosen to model raised ICP in this study.

### Kaolin injection resulted in increased intracranial pressure and ventricular dilatation

**Figure 2:**
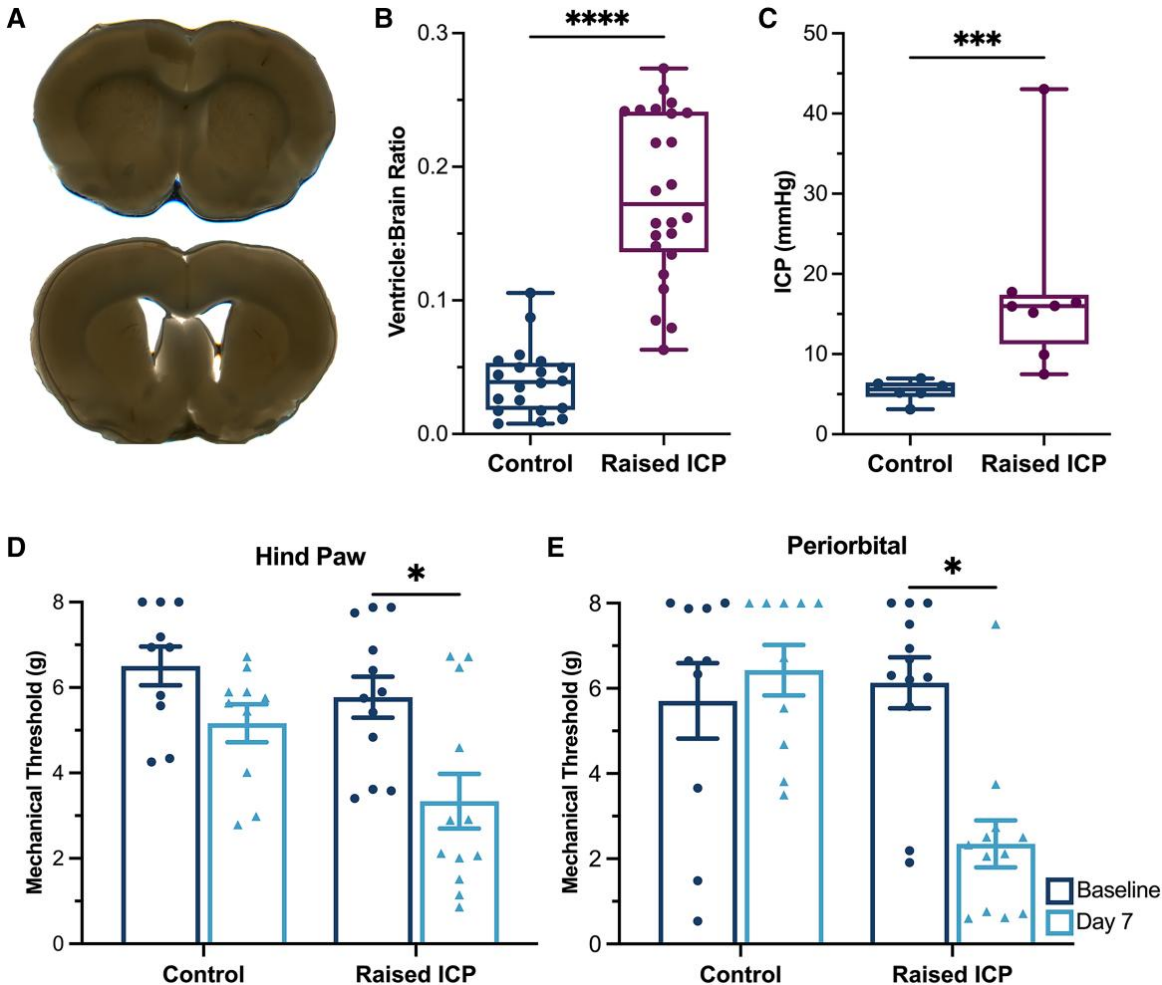
**Kaolin induced ventricular dilatation, raised intracranial pressure and reduced hind paw and periorbital mechanical thresholds.** (**A**) Coronal slices from control (saline: *top*) and raised intracranial pressure (ICP) animals (kaolin: *bottom*) demonstrating ventricular dilatation in those with raised ICP. (**B**) The ventricle:brain ratio was significantly higher in animals with raised ICP. (**C**) ICP was significantly increased in kaolin-injected animals compared with controls. (**D**) Hind paw mechanical withdrawal thresholds were significantly reduced in raised ICP animals at Day 7 compared with baseline. (**E**) Periorbital mechanical thresholds were also significantly reduced at Day 7 in raised ICP animals. Ventricle:brain ratio and ICP were analysed using Mann–Whitney U-testing; mechanical thresholds were tested using paired two-tailed *t*-tests. *P < 0.05, ***P < 0.001 and ****P < 0.0001.

Ventricular dilatation was used to confirm successful induction of hydrocephalus (Fig. 2A). The ventricle:brain ratio was significantly higher in animals with raised ICP [median (range) 0.17 (0.21), *n* = 24] compared with controls [0.04 (0.10), *n* = 20 (95% CI −0.1789 to −0.1008), *P* < 0.0001; Fig. 2B]. Epidural ICP was measured in a subgroup of animals injected with 40 µl of kaolin (raised ICP) versus an equal volume saline (control). ICP was significantly higher in the kaolin-injected animals versus controls [median (range) kaolin: 15.96 (8.97) mmHg, *n* = 8, control: 6.02 (1.79) mmHg, *n* = 6 (95% CI −12.89 to −4.368), *P* = 0.0007; Fig. 2C].

### Animals with raised intracranial pressure exhibited reductions in mechanical thresholds

Hind paw thresholds did not differ in control animals between baseline and Day 7 [baseline: 6.51 (1.44) g, Day 7: 5.16 (1.40) g, *n* = 10, *P* = 0.106; Fig. 2D]. In animals with raised ICP, hind paw mechanical thresholds were significantly reduced at Day 7 [baseline: 5.78 (1.66) g, Day 7: 3.34 (2.22) g, *n* = 12, *P* < 0.001; Fig. 2D]. Periorbital thresholds also did not differ in controls between baseline and Day 7 assessments [baseline: 5.71 (2.81) g, Day 7: 6.43 (1.88) g, *n* = 10; Fig. 2E). However, periorbital thresholds were significantly lower at Day 7 compared with baseline in animals with raised ICP [baseline: 6.13 (2.07) g, Day 7: 2.35 (1.91) g, *n* = 12, *P* < 0.001; Fig. 2E]. These studies demonstrate that animals exhibited changes in mechanical sensitivity following 7 days of exposure to raised ICP.

### Evoked spreading depolarization responses are altered in animals with raised intracranial pressure

**Figure 3:**
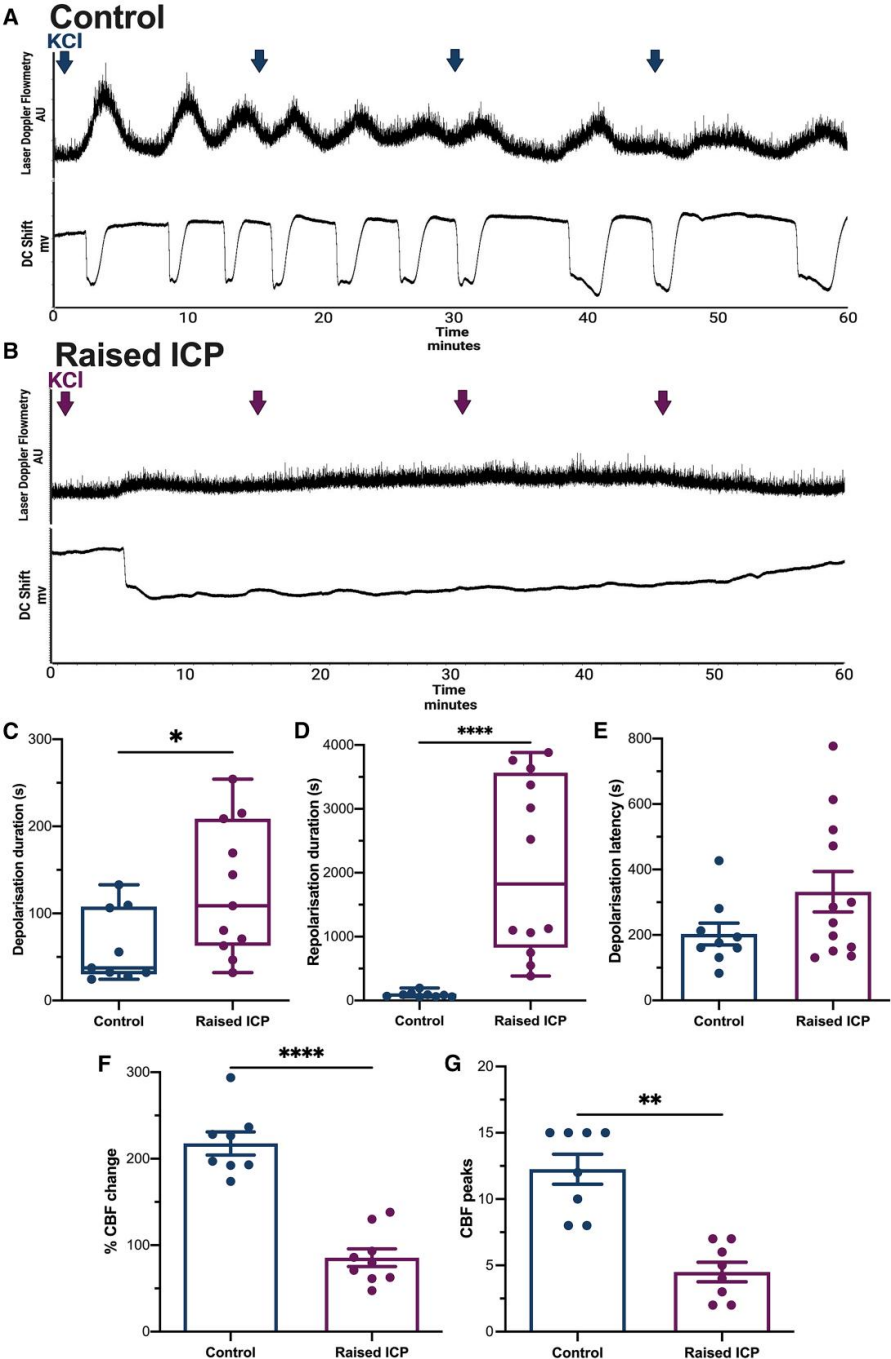
**Evoked spreading depolarization responses were drastically altered in animals with raised intracranial pressure.** (**A** and **B**) Representative steady-state potential (direct current, DC) and cerebral blood flow (CBF) response to evoked spreading depolarization in control (**A**) and raised intracranial pressure (ICP) animals (**B**). Arrows indicate when 5 μl KCl was added to the cotton pellet every 15 min. (**C**) Depolarization duration was increased in raised ICP animals. (**D**) Repolarization duration was increased in raised ICP animals. (**E**) Depolarization latency was similar between control and raised ICP animals. (**F**) Percentage change in CBF was lower in animals with raised ICP. (**G**) The number of CBF peaks within the 1 h recording was significantly lower in animals with raised ICP. Depolarization and repolarization durations were compared using Mann–Whitney U-testing; all remaining parameters were tested using unpaired two-tailed *t*-tests. *P < 0.05, **P < 0.01 and ****P < 0.0001. AU = arbitrary units.

We measured evoked spreading depolarization responses in animals with raised ICP. In control animals, cortical stimulation with KCl induced a rapid decline in the DC signal, which was coupled with an increase in CBF, characteristic of a typical spreading depolarization response (Fig. 3A).

Evoked spreading depolarization responses were drastically altered in animals with raised ICP (Fig. 3B). The duration of depolarization was significantly increased in animals with raised ICP [median (range) 108.81 (222.12) s, *n* = 11] compared with controls [37.54 (108.38) s, *n* = 9, *P* = 0.038 (95% CI 4.082 to 137.0); Fig. 3C]. The duration of repolarization was also markedly increased in animals with raised ICP [median (range) raised ICP: 1824.26 (3499.54) s, *n* = 12, control: 86.96 (140.05) s, *n* = 9, *P* < 0.0001 (95% CI 692.1 to 3435); Fig. 3D]. There was a loss of neurovascular coupling, because cortical tissue exhibited a significantly reduced change in CBF during depolarization [mean (SD) raised ICP: 85.55 (30.84)%, *n* = 9, controls: 217.64 (37.70)%, *n* = 8, *P* < 0.0001; Fig. 3F]. During the 1 h recording, animals with raised ICP exhibited a lower number of CBF peaks [mean (SD) raised ICP: 5 (2), *n* = 8] compared with controls [12 (3), *n* = 8, *P* = 0.005; Fig. 3G]. Evoked spreading depolarization responses in animals with raised ICP were markedly altered, exhibiting a delayed hyperpolarization following initial depolarization and a loss of neurovascular coupling.

### Energetic metabolites are altered in animals with raised intracranial pressure

**Figure 4:**
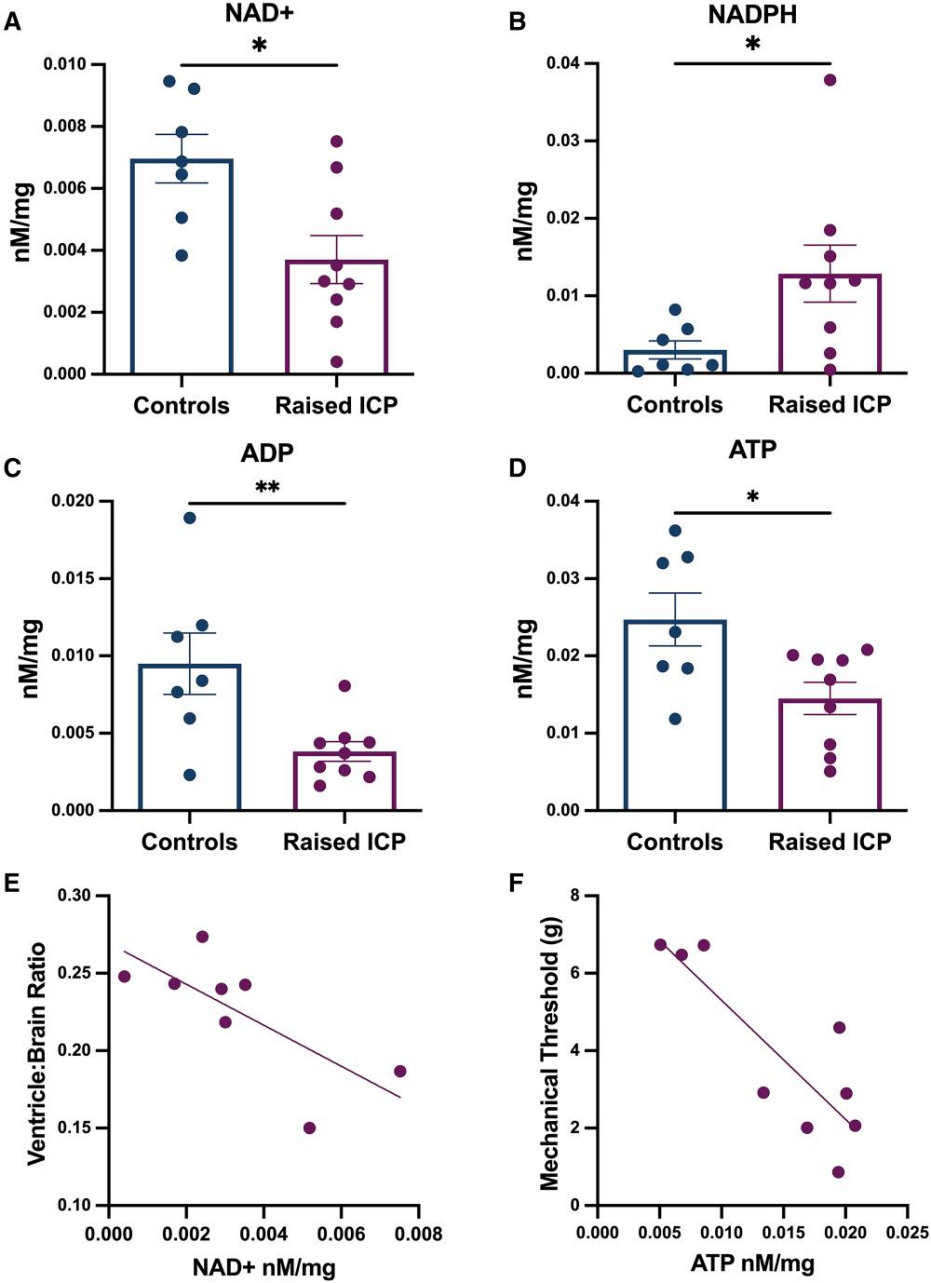
**Energetic metabolites were altered in the brains of animals with raised intracranial pressure.** (**A**) NADPH was increased in animals with raised intracranial pressure (ICP). (**B**–**D**) ADP (**B**), NAD^+^ (**C**) and ATP (**D**) were significantly reduced in animals with raised ICP. (**E**) NAD^+^ was correlated with ventricle:brain ratios, indicating that those with more ventricular dilatation had lower NAD^+^ concentrations (*r* = −0.7328, *P* = 0.039). (**F**) Hind paw mechanical thresholds at Day 7 also exhibited a correlation with ATP concentrations (*r* = −0.8428, *P* = 0.004). Metabolites were compared using unpaired two-tailed *t*-tests. *P < 0.05 and **P < 0.01.

Metabolites were extracted from whole-brain samples and compared between animals with raised ICP and controls. NADPH is crucial for maintaining the antioxidant capacity of cells and was found to be increased in animals with raised ICP [mean (SD) raised ICP: 0.0129 (0.0110) nM/mg, *n* = 9, controls: 0.0030 (0.0031) nM/mg, *n* = 7, *P* = 0.013; Fig. 4A] ADP, ATP and NAD^+^ measurements provide a readout of the energy status of cells and were all significantly lower in animals with raised ICP in comparison to controls [ADP, raised ICP: 0.0038 (0.0019) nM/mg, control: 0.0095 (0.0053) nM/mg, *P* = 0.009; Fig. 4B; NAD^+^, raised ICP: 0.0037 (0.0023) nM/mg, control: 0.0070 (0.0021) nM/mg, *P* = 0.011; Fig. 4C; ATP, raised ICP: 0.0145 (0.0062) nM/mg, control: 0.0247 (0.0091) nM/mg, *P =* 0.018; Fig. 4D]. In animals with raised ICP, a negative association was identified between NAD^+^ and ventricle:brain ratio (*r* = −0.7328, *P* = 0.039; Fig. 4E) and between ATP and hind paw mechanical thresholds (*r* = −0.8428, *P* = 0.004; Fig. 4F). These findings indicate that elevated ICP compromises energy metabolism and might relate to structural and behavioural features of the raised ICP model.

### GLP-1R agonism reduced intracranial pressure

**Figure 5:**
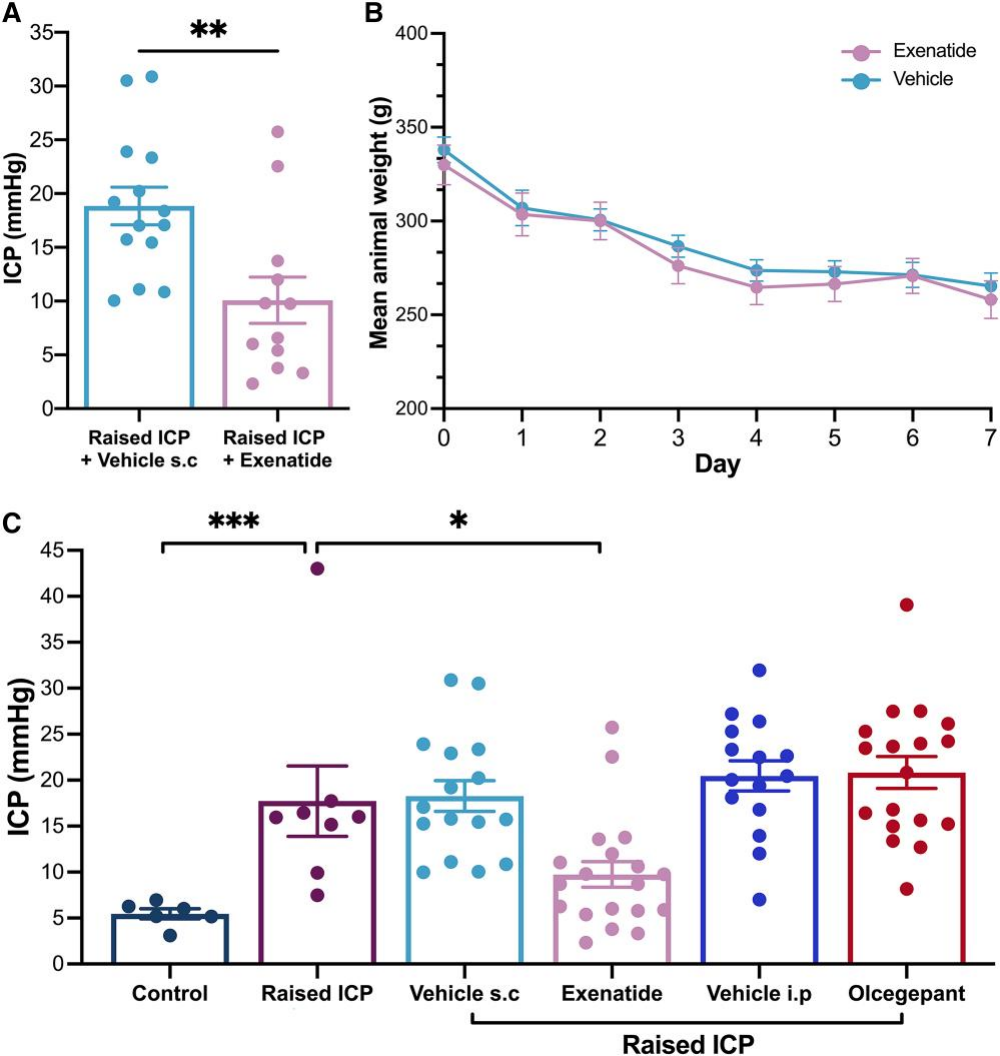
**The effects of GLP-1R agonist and CGRP-R antagonist on intracranial pressure (ICP) and weight in animals with raised ICP.** (**A**) ICP was lower in animals treated with GLP-1R agonist versus vehicle. (**B**) The weight of animals with raised ICP was similar between those treated with GLP-1R agonist and vehicle over 7 days. (**C**) ICP in controls and in animals with raised ICP in the absence of any intervention, in addition to GLP-1R agonist, CGRP-R antagonist and vehicle-treated subcutaneously (s.c,) or intraperitoneally (i.p.). ICPs were compared using unpaired two-tailed *t*-tests. *P < 0.05, **P < 0.01 and ***P < 0.0001. CGRP-R = calcitonin gene-related peptide receptor.

We investigated the impact of GLP-1R agonism on ICP, cortical excitability and mechanical withdrawal thresholds. In animals with raised ICP, daily administration of GLP-1R agonist resulted in significantly lower ICP [mean (SD) 9.74 (6.09) mmHg, *n* = 19] compared with vehicle-treated animals [18.27 (6.67) mmHg, *n* = 16, *P* = 0.004; Fig. 5A]. ICP was lowered within the range of control ICP values (Fig. 5C). Ventricular dilatation was identified in animals treated with vehicle and in those treated with GLP-1R agonist and did not differ significantly between groups (Supplementary Fig. 2). Weight change was similar between animals with raised ICP treated with vehicle versus GLP-1R agonist (Fig. 5B), suggesting that changes in ICP were independent of weight loss.

We did not find an effect of CGRP-R antagonism on ICP [mean (SD) CGRP-R antagonist: 20.84 (7.36) mmHg, *n* = 18, vehicle: 20.47 (6.37) mmHg, *n* = 15; Fig. 5C]. Therefore, CGRP blockade did not impact ICP in a model of raised pressure, a relationship that had not been explored previously.

### GLP-1R agonist rescued changes in hind paw and periorbital mechanical thresholds

**Figure 6:**
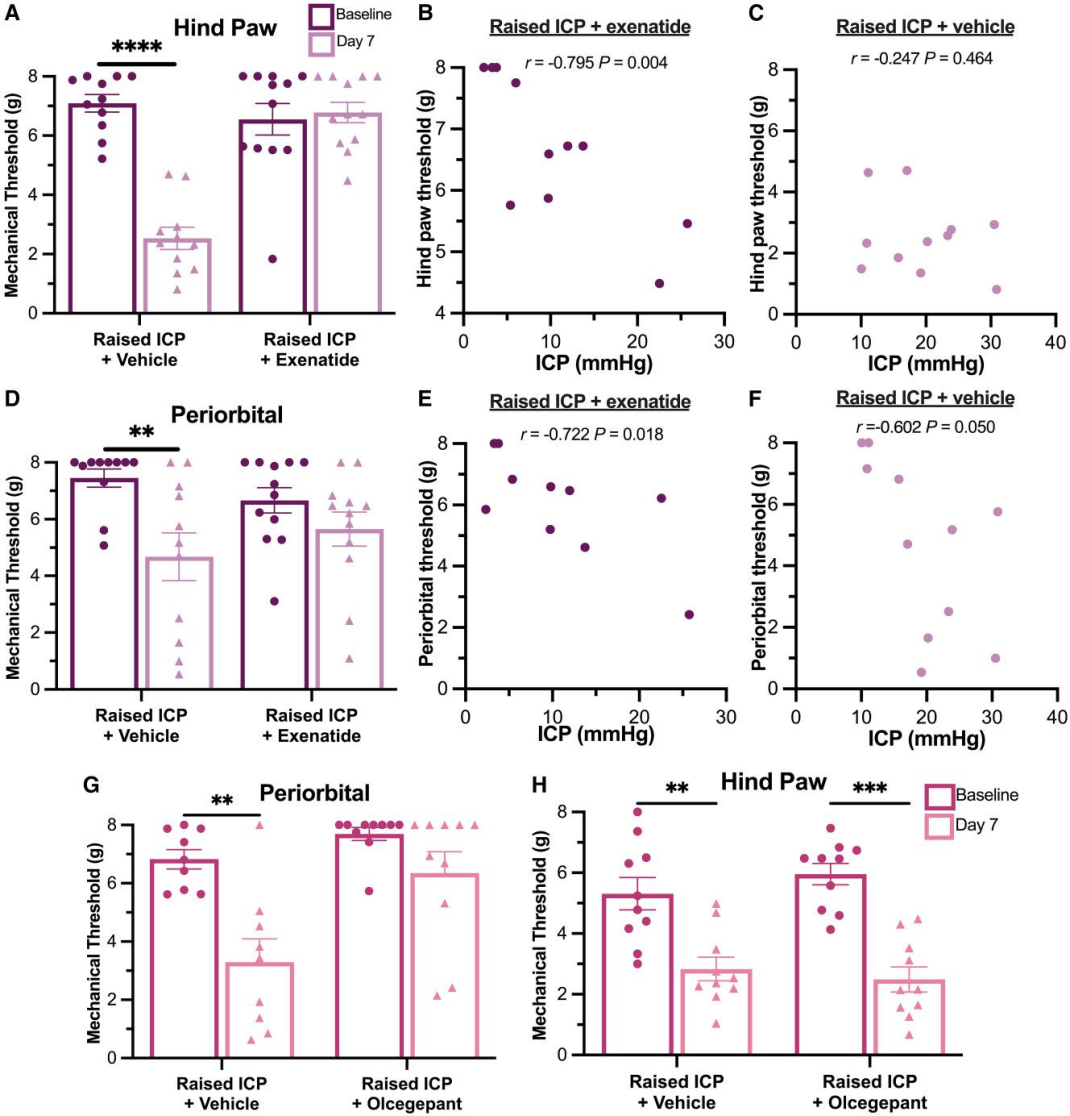
**GLP-1R agonist prevented changes in periorbital and hind paw mechanical withdrawal thresholds.** (**A**) Hind paw thresholds were significantly reduced at Day 7 in rats with raised intracranial pressure (ICP) treated with vehicle but not GLP-1R agonist. (**B** and **C**) Association between hind paw thresholds and ICP in rats treated with GLP-1R agonist (**B**) or vehicle (**C**). (**D**) Periorbital thresholds were significantly reduced at Day 7 in rats treated with vehicle but not GLP-1R agonist. (**E** and **F**) Periorbital thresholds were significantly associated with ICP in rats treated with GLP-1R agonist (**E**) and vehicle (**F**). (**G**) Periorbital mechanical thresholds were reduced in rats with raised ICP treated with vehicle but not CGRP-R antagonist. (**H**) Hind paw thresholds were reduced at Day 7 animals treated with vehicle and with CGRP-R antagonist. Mechanical withdrawal thresholds were compared using paired two-tailed *t*-tests. **P < 0.01, ***P < 0.001 and **** = *P <* 0.0001. CGRP-R = calcitonin gene-related peptide receptor.

Mechanical thresholds were assessed in naïve animals at baseline and at Day 7 following induction of raised ICP and daily injection of vehicle or GLP-1R agonist. Hind paw thresholds were reduced at Day 7 in the vehicle group [mean (SD) baseline: 7.15 (0.95) g, Day 7: 2.99 (1.97) g, *n* = 11, *P* < 0.0001; Fig. 6A]. In comparison, there were no differences in hind paw thresholds in the GLP-1R agonist-treated group [baseline: 6.36 (1.92) g, Day 7: 6.86 (1.22) g, *n* = 12]. There was a negative correlation between the ICP of animals treated with GLP-1R agonist and hind paw thresholds (*r* = −0.795, *P* = 0.004; Fig. 6B). Periorbital thresholds were reduced at Day 7 in the vehicle group [baseline: 7.02 (1.78) g, Day 7: 4.95 (2.84) g, *n* = 11, *P* < 0.01; Fig. 6D]. Rats treated with GLP-1R agonist, in contrast, did not exhibit changes in periorbital thresholds [baseline: 6.98 (1.11) g, Day 7: 5.69 (2.17) g, *n* = 12]. Both vehicle and GLP-1R agonist groups demonstrated a negative association between ICP and periorbital thresholds at Day 7 (GLP-1R agonist: *r* = −0.722, *P* = 0.018; Fig. 6E; vehicle: *r* = −0.602, *P* = 0.050; Fig. 6F). Therefore, reduction of ICP with GLP-1R agonist rescued periorbital and hind paw mechanical thresholds.

### CGRP-R antagonism prevented changes in periorbital but not hind paw thresholds in raised ICP

Periorbital thresholds were decreased in vehicle-treated animals [baseline: 6.83 (1.00) g, Day 7: 3.29 (2.39) g, *n* = 9, *P* = 0.003; Fig. 6G]. In comparison, animals with raised ICP treated with CGRP-R antagonist did not demonstrate differences between mechanical thresholds at baseline and Day 7 [baseline: 7.69 (0.71) g, Day 7: 6.35 (2.32) g, *n* = 10]. Animals with raised ICP treated with vehicle exhibited a significant decrease in hind paw thresholds at Day 7 compared with baseline [mean (SD) baseline: 5.31 (1.68) g, Day 7: 2.83 (1.23) g, *n* = 9, *P* < 0.01; Fig. 6H]. However, hind paw thresholds were also reduced at Day 7 in animals treated with CGRP-R antagonist [baseline: 5.96 (1.12) g, Day 7: 2.49 (1.30) g, *n* = 10, *P* < 0.0001]. These results reveal that CGRP-R antagonism was effective at preventing changes in periorbital thresholds in the setting of raised ICP.

### Reducing intracranial pressure restored spreading depolarization responses

**Figure 7:**
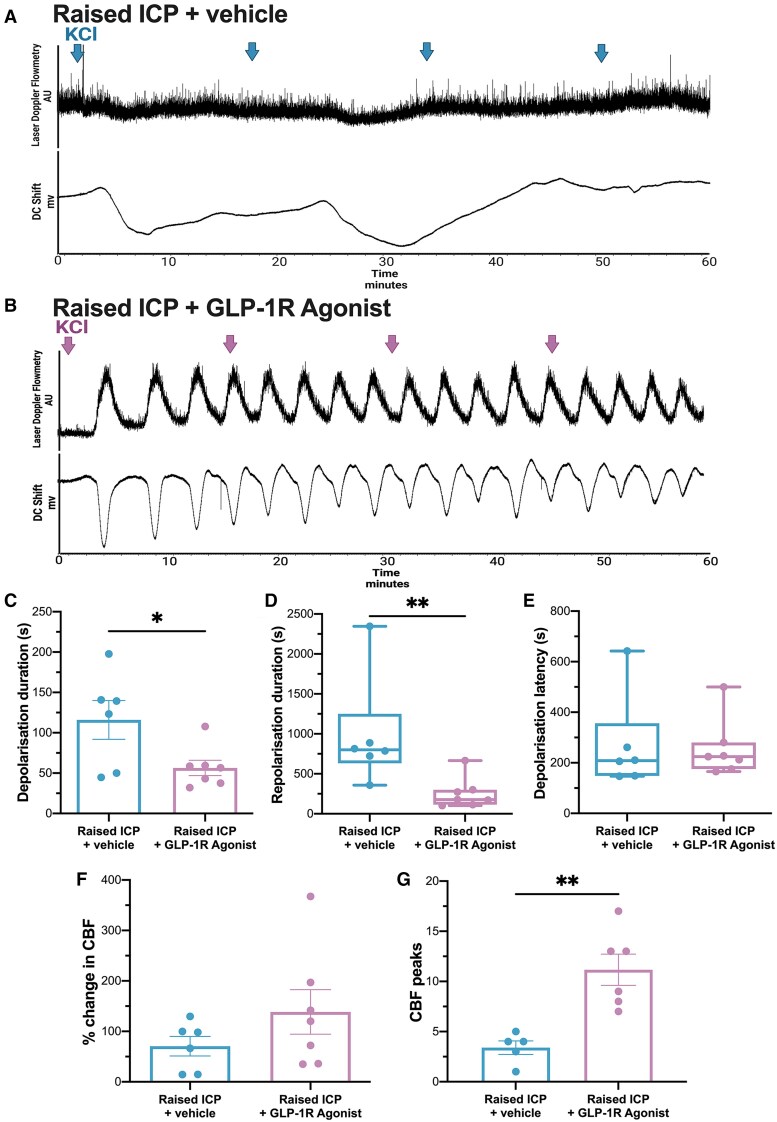
**GLP-1R agonist restored cortical and cerebral blood flow responses to spreading depolarization in animals with raised intracranial pressure.** (**A** and **B**) Representative direct current (DC) and cerebral blood flow (CBF) spreading depolarization response in animals with raised intracranial pressure (ICP) treated with vehicle (**A**) and GLP-1R agonist (**B**). Arrows indicate when 5 μl KCl was added to the cotton pellet every 15 min. (**C**) Depolarization duration was reduced following GLP-1R agonist treatment. (**D**) Repolarization duration showed a trend towards reduction in GLP-1R agonist-treated animals. (**E**) Depolarization latency was similar between vehicle and GLP-1R agonist-treated animals. (**F**) Percentage change in CBF. (**G**) The number of CBF peaks was higher in GLP-1R agonist-treated animals. Repolarization duration and depolarization latency were compared using Mann–Whitney U-testing; all remaining parameters were tested using unpaired two-tailed *t*-tests. *P < 0.05 and **P < 0.01. AU = arbitrary units.

In vehicle-treated animals, spreading depolarization responses were similar to those demonstrated by untreated animals with raised ICP (Fig. 7A). Treatment with GLP-1R agonist recovered cortical function and neurovascular coupling, with responses similar to those recorded from control animals (Fig. 7B). The duration of depolarization was significantly reduced [mean (SD) 56.46 (25.10) s, *n* = 7] compared with animals with raised ICP injected with vehicle [115.98 (58.80) s, *n* = 6, *P* = 0.033; Fig. 7C]. The duration of repolarization was also markedly decreased, because DC values returned to baseline more quickly [median (range) GLP-1R agonist: 177.55 (562.88) s, *n* = 7, vehicle: 800.85 (1988.67) s, *n* = 6, *P* = 0.002 (95% CI −1681 to −180.4); Fig. 7D]. CBF responses were improved with GLP-1R agonism, although this did not reach significance [GLP-1R agonist: 138.50 (108.14)%, *n* = 7, vehicle: 70.62 (47.75)%, *n* = 6, *P* = 0.212]. The number of CBF peaks was significantly higher in animals with raised ICP treated with GLP-1R agonist versus vehicle [mean (SD) GLP-1R agonist: 11 (4), *n* = 6, vehicle: 3 (2), *n* = 5, *P* = 0.0021; Fig. 7G]. These results demonstrated that GLP-1R agonism was able to restore typical evoked spreading depolarization responses in a model of raised ICP.

### Spreading depolarization responses were not altered by CGRP-R antagonist

CBF and neural spreading depolarization responses were similar between animals with raised ICP treated with vehicle or CGRP-R antagonism. Moreover, both the duration and the latency of depolarization were also not significantly different between animals treated with vehicle and CGRP-R antagonist [duration, vehicle: 203.06 (225.79) s, *n* = 5, CGRP-R antagonist: 162.17 (115.35) s, *n* = 9, latency, vehicle: 148.81 (50.25) s, *n* = 5, CGRP-R antagonist: 274.53 (190.70) s, *n* = 8]. Both groups exhibited a lag in repolarization [mean (SD) vehicle: 1503.19 (1132.18) s, *n* = 5, CGRP-R antagonist: 1561.23 (1206.01) s, *n* = 9]. Moreover, CGRP-R antagonist did not improve neurovascular responses [mean (SD) CBF change, vehicle: 69.02 (72.68)%, *n* = 5, CGRP-R antagonist: 73.06 (42.66)%, *n* = 9]. The number of CBF peaks was also not different between animals with raised ICP treated with vehicle or CGRP-R antagonist [mean (SD) vehicle: 3 (2), *n* = 5, CGRP-R antagonist: 4 (2), *n* = 9]. In summary, CGRP antagonism did not impact cortical or neurovascular spreading depolarization responses in the setting of raised ICP.

## Discussion

The relationship between cephalic pain and cerebrovascular changes in the setting of raised ICP is poorly understood. These data suggest that elevated ICP disrupts cortical neurovascular responses, causes cephalic allodynia and depletes crucial energetic substrates. GLP-1R agonism effectively reduced ICP, which led to improved pain thresholds and cortical neurovascular function. Blocking CGRP with olcegepant alleviated cephalic pain, indicating a role for CGRP in mediating pain responses associated with raised ICP. These findings support the rationale that reducing ICP can alleviate cephalic pain in conditions of raised ICP. Moreover, the data suggest that headaches associated with elevated ICP might be managed therapeutically by blocking the CGRP pathway.

### Neurovascular responses are altered in raised intracranial pressure

In models of raised ICP, changes in cerebral perfusion pressure have previously been studied and proved to be abnormal.^35^ However, this study progresses this knowledge by evaluating cerebrovascular changes coupled with dynamic changes in spreading depolarization. We have used spreading depolarization as a marker for changes in cortical hyperexcitability. Spreading depolarization is important because it is observed in conditions with raised ICP.^7^ We have shown, for the first time, dramatic uncoupling of the neurovascular response to spreading depolarization.

We have also demonstrated that CBF responses to evoked spreading depolarization were altered in raised ICP and did not exhibit characteristic CBF changes during spreading depolarization. Animal models of traumatic brain injury have previously identified a negative correlation between ICP and CBF.^36^ At high ICP and cerebral perfusion pressure, vessels can be fully constricted or dilated, hampering local vascular responses to neural activity.^37^ Although we have noted altered cerebrovascular responses in raised ICP, which is of interest, we would not suggest that alterations in spreading depolarization are a measure indicative of cephalic pain.^38^ The implications of changes in spreading depolarization are not fully understood and would need future exploration.

### Cellular energetic substrates depleted in raised intracranial pressure

We also found that metabolites associated with key indicators of cellular energy status, including ADP, ATP and NAD^+^, were all significantly lower in raised ICP. Although these substrates for cellular energetics have not been evaluated previously, our findings corroborate studies of brain microdialysis in patients with hydrocephalus and brain injury that identified downstream metabolite signatures suggestive of impaired cellular energy metabolism.^21,39,40^ Lacking availability of energetic substrates might explain the impaired recovery during spreading depolarization noted in these animals with raised ICP. It is interesting to speculate that supplementing energetic substrates could enhance recovery of the spreading depolarization. For example, magnesium supplementation, which contributes to metabolic pathways that synthesize and recycle NAD^+^, has been shown to increase tissue resistance to spreading depolarization.^41^ Coenzyme Q10 is an integral component of the mitochondrial electron transfer chain responsible for biosynthesis of ATP and NADH, and supplementation has been shown to improve migraine burden.^42,43^ It would be interesting to speculate that supplementation in a raised ICP model, where we have shown that ATP and NAD^+^ are lacking, would facilitate recovery of spreading depolarization and cephalic pain. Interestingly, we observed that NADPH was elevated in raised ICP; this is an antioxidative metabolite and is likely to indicate a compensatory mechanism.^44^

### GLP-1R agonism reduced intracranial pressure in a rodent model

Emerging clinical studies have demonstrated the ability of exenatide to reduce ICP.^22^ In animals with raised ICP, we found that exenatide returned ICP to levels similar to control animals. This emphasizes the therapeutic potential of exenatide in patients with conditions associated with raised ICP.

GLP-1R agonists are known to modify appetite and satiety and can lead to weight loss, and some GLP-1R agonists, such as semaglutide, are used as treatments for obesity.^45^ However, we observed no differences in weight between animals receiving exenatide versus vehicle, suggesting that the reduction in ICP is unlikely to be explained by weight loss.

### Reducing intracranial pressure improved corticovascular responses

We went on to reduce ICP with a GLP-1R agonist and show recovery of the corticovascular response. In animals with normal ICP, GLP-1R agonism did not improve the corticovascular response. This suggests that it is the reduction in ICP that improves the corticovascular response, not the drug itself. This is important to distinguish, because in clinical studies GLP-1R agonists have been shown to increase tissue blood flow in skeletal and cardiac muscle.^46^ Of interest, the GLP-1R agonist exenatide has been shown to increase tissue oxygenation in rodent models, indicating additional potentially beneficial effects of the drug in the CNS.^47^

### Reducing intracranial pressure was effective at improving allodynia

Our study has illustrated that in the setting of raised ICP and altered neurovascular responses, there is an increase in cutaneous allodynia. This has not been demonstrated previously in animal models. However, these findings are supported by clinical observations that, in diseases with raised ICP, headache is a common clinical feature. For example, 95% of idiopathic intracranial hypertension patients report headache,^48^ post-traumatic headache is commonly observed following traumatic brain injury,^4^ and headache is a cardinal sign in stroke^8^ or for a tumour with associated raised ICP.^49^ Patients with raised ICP have demonstrated a high prevalence of chronic pain disorders,^50^ relating to our finding of reduced hind paw mechanical thresholds. In humans, it is also recognized that back pain (53%), neck pain (42%) and radicular pain (19%) occur in the setting of raised ICP, potentially relating to compression of nerve roots from the elevated ICP.^51^

Reducing ICP with a GLP-1R agonist was effective at improving both cephalic and extra-cephalic allodynia in our model. Furthermore, the degree of allodynia was correlated with the level of raised ICP. This indicates the association between raised ICP and cephalic allodynia. This is of relevance because in humans with raised ICP and headaches, there is debate concerning the role of raised ICP in driving allodynia and additional debate as to whether reducing ICP can improve headache.^3,52^ Our animal study suggests that reducing ICP is likely to have an impact on cerebral nociception. The exact mechanisms by which reducing ICP improves pain behaviours, however, are unclear.

### CGRP-R antagonism improved cephalic allodynia in the setting of raised intracranial pressure

In the presence of elevated ICP, inhibiting CGRP improved cephalic allodynia. Our results imply that CGRP signalling might play a role in cranial nociception in the setting of raised ICP. CGRP-R antagonism has been shown to attenuate nociception in animal models of cephalic pain.^32,53-55^ Our results indicate that CGRP signalling is likely to be involved in cephalic pain attributable to raised ICP, in addition to the therapeutic potential of blocking CGRP in raised ICP. We note that olcegepant, the CGRP-R antagonist, did not affect ICP directly, indicating that its therapeutic efficacy to modulate allodynia was likely to be a direct effect on nociceptive pathways and not attributable to effects on ICP itself.

### Limitations and future directions

Our present study has some limitations. Although it is known that spreading depolarization is observed across various clinical conditions associated with raised ICP,^36,56-60^ only some of these conditions display features of aura, which is felt to be a clinical correlate of spreading depolarization. It is noted in patients with idiopathic intracranial hypertension that 17–40% have migraine with aura,^3,51^ likewise in post-traumatic headache (2.2–50%)^61-64^ and stroke (3.6–12.5%).^65-67^ However, aura represents only one aspect of the broader corticovascular disturbances associated with spreading depolarization in the setting of raised ICP. It is known that mechanical thresholds and CGRP expression display sexual dimorphism,^68^ hence given that our studies were conducted only in male rats, the generalizability to female rats cannot be determined. Additionally, spreading depolarization is known to be highly variable depending on the oestrus cycle phase,^25^ and this would be of interest to evaluate in the future. Anaesthetic agents can impact spreading depolarization, and it is possible that the use of propofol in these experiments would have altered the cerebral corticovascular responses.^69^ However, to ensure comparability of results, both treatment groups were maintained under the same anaesthesia. Our study is not modelling any discrete clinical condition but is of relevance to understanding the physiology of raised ICP, which might be applicable to multiple conditions, including traumatic brain injury, stroke, subarachnoid haemorrhage and idiopathic intracranial hypertension.

## Conclusion

In summary, our study demonstrates the impact of elevated ICP on cerebral vascular changes and allodynia. In the setting of raised ICP, we observed disrupted cerebral vascular function and responses to spreading depolarization, with impaired neurovascular coupling and depletion of energetic substrates. Reducing ICP with exenatide, a GLP-1R agonist, restored spreading depolarization and cerebral vascular responses. In addition, reducing ICP improved cephalic allodynia. Finally, blocking CGRP nociception in animals with raised ICP improved cranial allodynia, suggesting a role for CGRP in driving cephalic pain. Our results suggest two separate therapeutic pathways that can potentially ameliorate cephalic allodynia in the setting of raised ICP: (i) reducing ICP with GLP-1R agonism; and (ii) blocking CGRP. Translating our results into patients with raised ICP suggests that reducing ICP and blocking CGRP could be therapeutic for headache.

## Supplementary Material

awae415_Supplementary_Data

## Data Availability

The data that support the findings of this study are available from the corresponding author, upon reasonable request.
