# Supplementary material for: Raised intracranial pressure alters cortical vascular function and cephalic allodynia
Source: Brain. 2025 Mar 8;148(6):2163–77. doi: 10.1093/brain/awae415 (PMC12129729; doi:10.1093/brain/awae415)
Supplement: awae415_Supplementary_Data [file awae415_supplementary_data.pdf]

# Raised intracranial pressure alters cortical vascular function and cephalic allodynia.

**Authors:** Olivia Grech[1,2] Eloisa Rubio-Beltran [3], Emily C. Stanyer [3,4], Alejandro Labastida-Ramirez [3], Gareth G. Lavery [5], Lisa J. Hill [1,2], Philip R. Holland [3], and Alexandra J. Sinclair[1*].

**Affiliations:**

[1]Institute of Metabolism and Systems Research, College of Medical and Dental Sciences, University of Birmingham, Birmingham, B15 2TT, UK

[2]School of Biomedical Sciences, Institute of Clinical Sciences, University of Birmingham, Birmingham, B15 2TT, UK.

[3]Headache Group, Wolfson Sensory, Pain and Regeneration Centre, Institute of Psychiatry, Psychology and Neuroscience, King's College London, London, SE1 3RB, UK

[4]Sleep and Circadian Neuroscience Institute, Nuffield Department of Clinical Neurosciences, University of Oxford, Oxford, OX3 9DU, UK

[5]Centre for Systems Health and Integrated Metabolic Research, Department of Biosciences, School of Science and Technology, Nottingham Trent University, Clifton Campus, Nottingham, NG11 8NS, UK

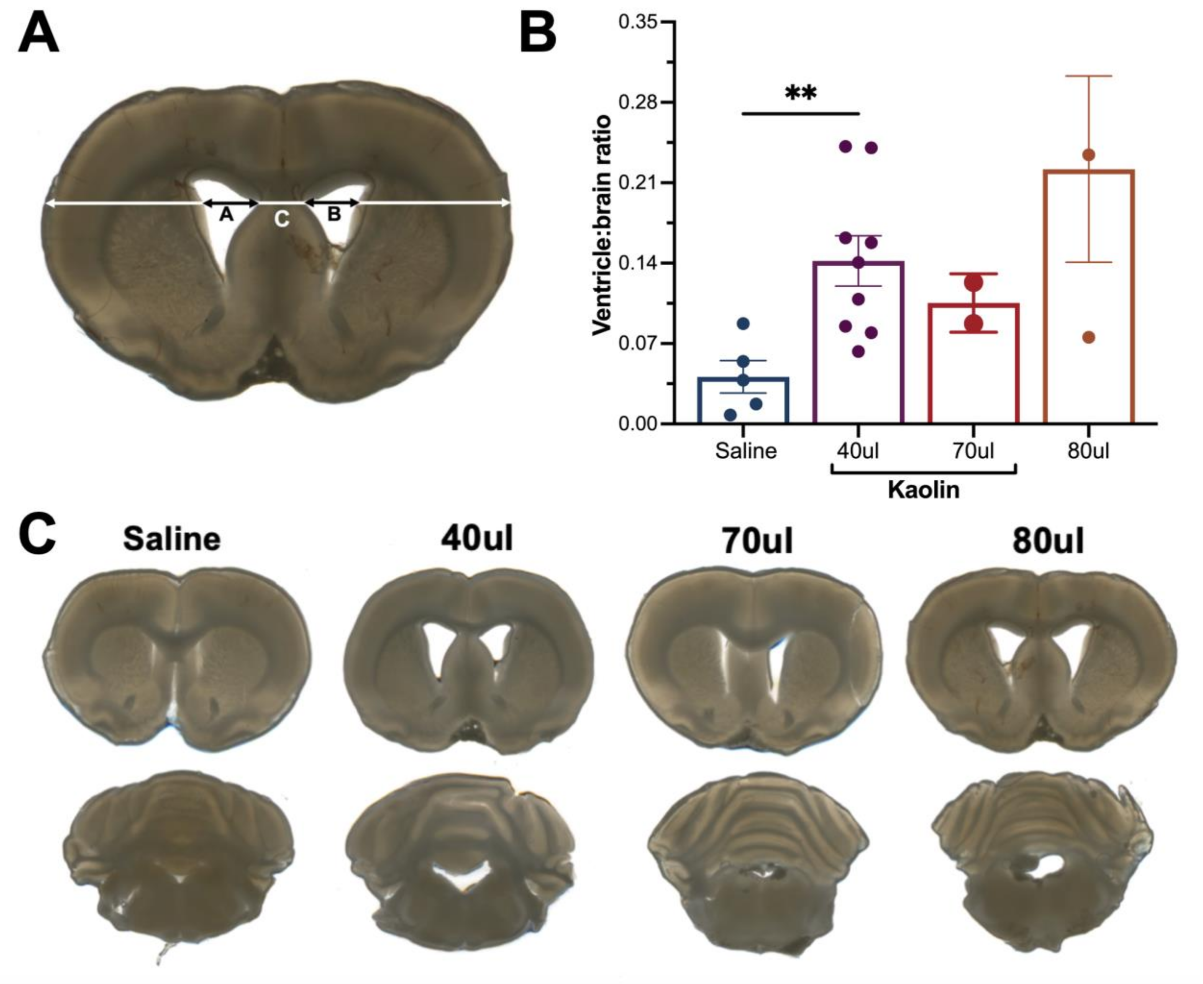


**Supplementary Fig 1. Pilot studies optimizing the volume of kaolin in the raised ICP model.** (**A**) Method of ventricle:brain ratio quantification. (**B**) Quantification of ventricle:brain ratio in pilot experiments. (**C**) Coronal brain regions of rodents injected with 40µl saline, 40µl, 70µl and 80µl of kaolin. (** = $p<0.01$)

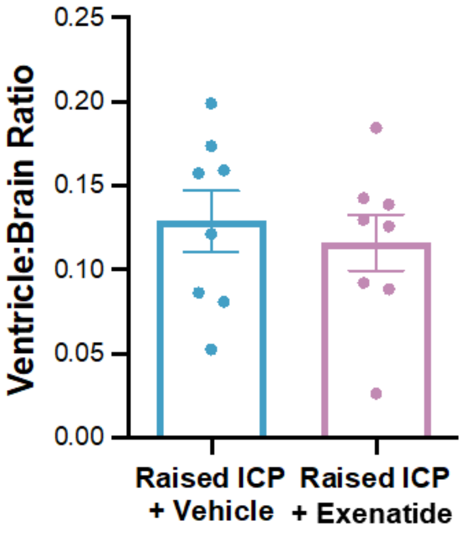


**Supplementary Figure 2. Ventricle:brain ratio is not significantly different between raised ICP animals treated with vehicle or exenatide.** (ICP; intracranial pressure).
